# Introducing a Method for Intervals Correction on Multiple Likert Scales: A Case Study on an Urban Soundscape Data Collection Instrument

**DOI:** 10.3389/fpsyg.2020.602831

**Published:** 2021-01-22

**Authors:** Matteo Lionello, Francesco Aletta, Andrew Mitchell, Jian Kang

**Affiliations:** Institute for Environmental Design and Engineering, The Bartlett, University College London, London, United Kingdom

**Keywords:** multiple likert scales, ordinal against interval scales, likert scale correction, likert equidistance, urban soundscape, soundscape modeling, soundscape indices

## Abstract

Likert scales are useful for collecting data on attitudes and perceptions from large samples of people. In particular, they have become a well-established tool in soundscape studies for conducting *in situ* surveys to determine how people experience urban public spaces. However, it is still unclear whether the metrics of the scales are consistently interpreted during a typical assessment task. The current work aims at identifying some general trends in the interpretation of Likert scale metrics and introducing a procedure for the derivation of metric corrections by analyzing a case study dataset of 984 soundscape assessments across 11 urban locations in London. According to ISO/TS 12913-2:2018, soundscapes can be assessed through the scaling of 8 dimensions: pleasant, annoying, vibrant, monotonous, eventful, uneventful, calm, and chaotic. The hypothesis underlying this study is that a link exists between correlations across the percentage of assessments falling in each Likert scale category and a dilation/compression factor affecting the interpretation of the scales metric. The outcome of this metric correction value derivation is introduced for soundscape, and a new projection of the London soundscapes according to the corrected circumplex space is compared with the initial ISO circumplex space. The overall results show a general non-equidistant interpretation of the scales, particularly on the vibrant-monotonous direction. The implications of this correction have been demonstrated through a Linear Ridge Classifier task for predicting the London soundscape responses using objective acoustic parameters, which shows significant improvement when applied to the corrected data. The results suggest that the corrected values account for the non-equidistant interpretation of the Likert metrics, thereby allowing mathematical operations to be viable when applied to the data.

## 1. Introduction

Likert scales (Likert, 1932) are commonly used in social sciences for the collection of attitudes and opinions. A Likert scale is composed of an odd number (typically 5 or 7) of ordered categories ranging from “strongly disagree” to “strongly agree” (or vice versa) with a “neutral” assessment being the midpoint category. Each point in the scale represents the degree to which the respondent agrees or disagrees with regard to a specific statement or construct, which is then typically associated with a value. There has been a long debate (Jamieson, 2005; Pell, 2005; Carifio and Perla, 2008) around whether or not the categories of a Likert scales can be interpreted by people as being equidistant. In their original conception, Likert scales are a sorted sequence of ranked categories where only nonparametric tools can be used. Inferring that the scales have an equidistant property between their categories allows the use of more powerful and precise parametric tools, and potentially mathematical operations (Adroher et al., 2018). This assumption is called an interval interpretation of the scales. It is indeed common practice for researchers to assume that participants will interpret the categories in the scales as equidistant (Lionello et al., 2020). The case of multiple scales mapped to a low-dimensional space expands the challenge of validating the equidistance property, both within each scale and between different scales of the same instrument, as in the case of the soundscape data collection protocol considered in this study.

Performing a scaling task with a Likert instrument essentially means mapping a perceptual space. Thus, trying to validate the equidistance property with a separate experiment would be challenging as it would imply mapping a different space through a potentially nonidentical task (see section 2.2). For this reason, any attempt at validating equidistance of Likert categories should be sought within datasets originating from the same scaling task (Lantz, 2013).

**Figure 1:**
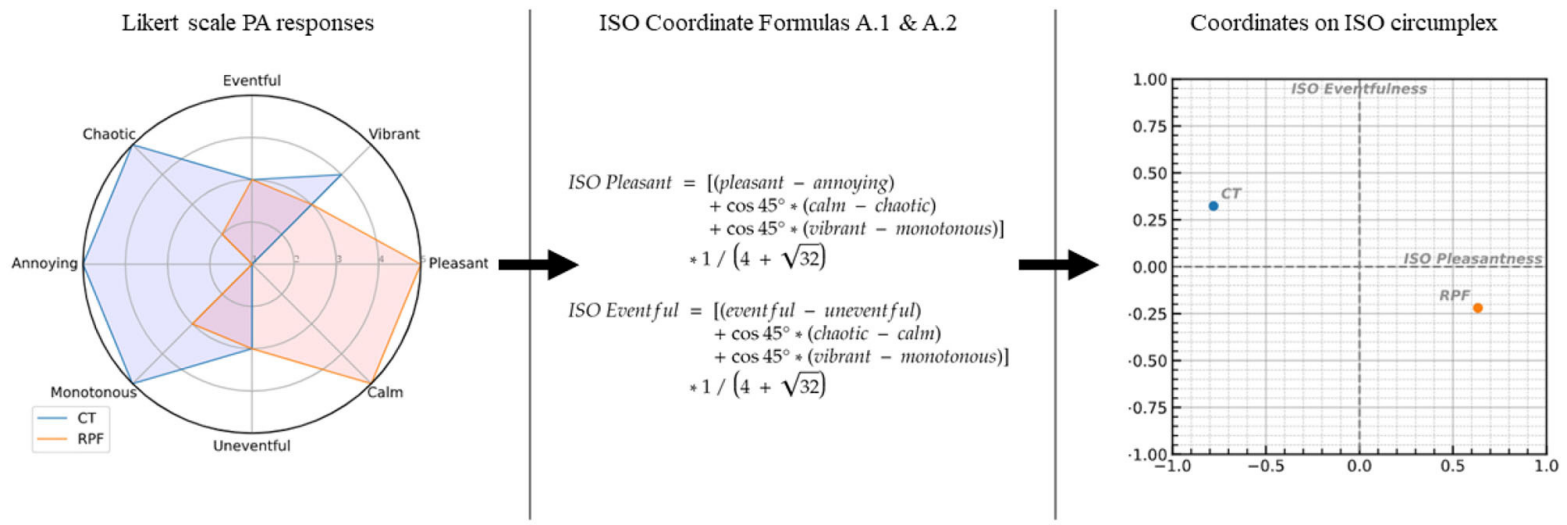
The procedure for projecting the 8 perceptual attribute dimensions **(left)** onto the bidimensional circumplex model **(right)** according to the formulas given in ISO12913:3-2019 **(center)**. The data used are example single surveys drawn from Camden Town (CT) and Regents Park Fields (RPF). Each line of the radar plot shown on the left represents one perceptual attribute laid onto the bidimensional space, with paired attributes in opposing directions. The transformation is performed according to formulas A.1 and A.2 given in ISO12913:3-2019. The $*1/\left(4+\sqrt{32}\right)$ term is included in order to scale the resulting coordinate values between −1 and +1.

Soundscape, which is defined as the perceived sound environment by individuals and people in context (ISO, 2014), is going through a standardization process, especially for data collection instruments and corresponding analysis techniques. Assessment scales (e.g., Likert scales, Visual Analog scales, etc.) play an important role in the development of methods and tools for soundscape analysis (Fiebig and Herweg, 2017; Aletta et al., 2019; Lionello et al., 2020). One of the procedures currently used for soundscape assessments is the “Method A” described in the ISO/TS 12913-2:2018 (see section 2.1). This method makes use of Likert scales as its primary tool; while originally defined for “soundwalks” (i.e., assisted listening exercises on site) that are typically designed for 10–20 participants, the Method A could also be used for large-scale soundscape surveys on site, enabling the collection of data from, potentially, hundreds of public spaces users in a relatively short period of time. Several adjectives, which the model by Axelsson et al. (2010) assumes to be laying onto a vector space where their correlation is known (see section 2.1 and Figure 1), are presented to participants for them to indicate their degree of agreement or disagreement on whether each adjective is suitable to describe the soundscape they experience. These adjectives, which in the context of this study are referred to as “perceptual attributes,” are defined to represent the dimensional components describing the decision process occurring in the quality evaluation of the soundscape experience by listeners. By assigning each category to a given number, certain assumptions introduced in section 2.1 allow researchers to mathematically collapse several scales into one or more values to describe the average assessment of the soundscape. The current study aims to understand the limits within which these operations can take place, and where correction factors can be placed in order to best report the abstract representation of urban soundscapes in the listener's mind.

In a previous study (Lionello et al., 2019), soundscape datasets were collected at different sites. Strong dependencies were found between the percentages of scores falling in three groups of Likert categories, i.e., “agreement,” “disagreement,” and “neutral,” across different locations for different perceptual attributes. Nonetheless, some soundscapes datasets were found to show an asymmetric distribution across the mean and variances of their perceptual attributes and their average was observed to fall on a larger interval compared to the average of the corresponding opposite attribute. These findings encouraged a larger and more systematic investigation of the interpretation of the metric scales, showing that the typical inference of an equidistant property may occasionally be violated. In the current study, the previous analysis is extended to a larger dataset: the general goal is determining a procedure that is potentially applicable to other soundscape studies for the introduction of scale correction values. To the best of the authors' knowledge, this study represents the first attempt in soundscape literature to apply psychometric correction factors to soundscape assessment scales.

The correction values found are bound to the paradigm defined by the data collection framework; although the overall methodology would remain valid, a change in (even small) aspects of the assessment task (as detailed in section 5.6) would likely render the derived correction values themselves invalid. In the current case, the model is bound to the following points: five-point Likert scales where each point is labeled; *in situ* data collection; and the scale selection to be assessed and the target of the scales assessments, while the conditions for which the current methodology can be applied are as follows: multiple Likert scales laying on a known vector space; multiple sets of situations where surveys are collected; and a consistent sample of surveys (*N*≈100) assessed for each of these sets. At this stage, under certain hypothesis discussed in section 5.2, the correction factors are assumed to be invariant with respect to the sample of soundscapes currently reported. The study aims to address two main research questions: Are the relationships between Likert categories in each scale and between scales coherently understood by participants as expected from the equidistant property of Likert scales? If not, what corrections can be introduced to adjust them and to project soundscapes assessment that are more consistent with the participant's interpretation?

In section 2, a theoretical background related to soundscape data collection, analysis standards, and Likert scaling task will be presented and the main issues are identified. In sections 3 and 4, the protocol followed for the data collection is introduced and the results of the method applied to soundscapes are reported. In section 5, the correction factors for the investigated metrics and the limitations of the proposed framework are discussed.

## 2. Theoretical Background for Application to Soundscape Studies

### 2.1. Introduction to the ISO 12913 Series on Soundscape

The International Organization for Standardization (ISO) has been working during the past decade on the ISO 12913 (Acoustics—Soundscape) series. This currently includes three parts: Part 1—definition and conceptual framework (ISO, 2014); Part 2—data collection and reporting requirements (ISO/TS, 2018); and Part 3—data analysis (ISO/TS, 2019). Part 1 is published as a full standard document, while the other two parts are published as technical specifications. Part 1 defines the “soundscape” as a perceptual construct, as opposed to the “acoustic environment,” which is the physical phenomenon. Parts 2 and 3 are the “operational” documents where the instruments for data collection and analysis procedures are described. Part 2 provides three different options for gathering data on how people experience(d) acoustic environments (i.e., soundscape data), which include questionnaires to be used on-site (Method A or Method B), and narrative interviews protocols to be used off-site (Method C). In this study, we focus on Method A; this is adapted from the previously established Swedish Soundscape Quality Protocol (SSQP), emerging from the work on urban soundscapes by Axelsson and colleagues at the University of Stockholm during the years 2005–2010 (Axelsson et al., 2010). The perceptual attributes used for the soundscape assessment were defined in the context of a laboratory experiment; they were selected from the analysis of the principal components across 116 adjectives scaled by 100 students on 50 audio-recordings of urban locations in Sweden (Axelsson et al., 2010). The experiment validated the circumplex model of affect (Russell, 1980; Posner et al., 2005) for soundscape assessment tasks and the following 8 perceptual attributes were identified: “pleasant,” “annoying,” “vibrant,” “monotonous,” “calm,” “chaotic,” “eventful,” and “uneventful.” The dimensions corresponding to the perceptual attributes lay onto a bidimensional space described in Figure 1 where pleasant and annoying are parallel to each other and orthogonal to eventful and uneventful, which are also parallel to each other. The other four perceptual attributes lay along the bisectors of the plan. Perceptual attributes that are parallel to each other can be gathered in four pairs: “pleasant-annoying,” “calm-chaotic,” “vibrant-monotonous,” and “eventful-uneventful.” The scaling of these eight perceptual attributes was included in the Method A of the ISO/TS 12913-2:2018, where the protocol requires the participants to listen to a given acoustic environment and then proposes the following task: “For each of the eight scales below, to what extent do you agree that the present surrounding sound environment is.,” followed by eight perceptual attributes, each associated to a Likert scale. This instrument had effectively been used in soundscape studies for several years before it was included in the ISO/TS 12913-2:2018, and until the ISO/TS 12913-3:2019 document was published, there was no clear indication on how the data collected through this protocol should have been analyzed or indeed “represented.” Simply plotting the mean scores of the participants' sample as individual values on the circumplex model was often considered a pragmatic approach (Aletta et al., 2017; Kang et al., 2018): using a spider plot would allow to visualize an average “soundscape profile” for a given acoustic environment (see Figure 1). However, this is not a particularly comprehensive representation, nor one that allows for easy and meaningful comparisons between soundscapes. Therefore, Part 3 of the ISO series offers further guidance; it provides that the 8 attributes should be projected onto the bidimensional circumplex model by computing an orthonormal projection (Kogan et al., 2016; Lindburg and Friberg, 2016; ISO/TS, 2019) onto the two main dimensions of the circumplex model, which from now on we will identify as “ISO Pleasantness” and “ISO Eventfulness” to distinguish them from the simple perceptual attributes. This process is schematized (Figure 1): it shows how the assessment of a soundscape derived from the eight attributes scored independently can be reported to a point (x = ISO Pleasant; y = ISO Eventful) on the ISO circumplex model. Such an orthonormal projection assumes the participants to interpret the categories of the single Likert scales as being equidistant, and the eight perceptual attributes to be related as per the circumplex model. By following this assumption, it is possible to match, for instance, disagreement of annoying with agreement of pleasant, and a neutral score of pleasant with a neutral score of annoying, and so on for all the paired perceptual attributes. Having a final pair of coordinates allows the soundscape to be pinned in the circumplex model in order to cluster agglomerations of soundscapes, to classify the soundscapes according to the perceptual attribute dictating the bisector of the quarter where they fall, and to calculate the distances between soundscapes and the distances from them and the axes. The introduction of redundancy in the scaling of all the eight perceptual attributes is supported by the idea that, during the scaling task, participants may focus their attention to different categories of sounds according to the valence of the sound source (Berglund et al., 2007). The scaling of all the eight perceptual attributes also introduces a higher resolution in the final projection and it could also be used as an exclusion criterion for those participants whose assessments fall too far from the overall statistics.

Although one could imagine the kind of soundscapes falling along the edge regions of each bisector (e.g., distant traffic noise for monotonous, sounds of urban parks for calm, festive alleys atmosphere for vibrant, street affected by loud traffic noise for chaotic, etc.), and so to gradually shift from one of these to another one, a potential problem is to understand what kind of soundscape location could be represented in the center of the circumplex model and what is the meaning of the distance from one point to the center. A second challenge is whether the model should be inscribed within a circle, as currently described in Part 3 of the ISO (ISO/TS, 2019), or rather inside a square and making so, for instance, the agreement of vibrant match the agreement of pleasant. Moreover, it is not possible to know if the dimensions maintain exact overlapping intervals and ranges between each other.

### 2.2. Scaling Task and Equidistance of Likert Scale Categories

Some factors inherent in an *in situ* survey (such as ecological validity, participants' psychological state and attention, behavioral and routine context, and variety of population) introduce deviations in the *in situ* scaling compared to a laboratory setup (Rickards et al., 2012). The introduction of these deviations means the *in situ* scaling task does not represent an endomorphism within the space originally found from the principal component analysis performed in the laboratory experiments. The mapping of the *N*-dimensional abstract representation of the soundscape in the participant's mind to the 8-dimensional space potentially affects the interpretation of the Likert scales without maintaining the assumed equidistance property between the points of the original field (Maffiolo et al., 1999). In this way, the scaling task may result in the negative and positive poles of the Likert scale being unbalanced, collapsing one edge, dilating the distance between points, or omitting middle points. Furthermore, the assumption of endomorphism would not justify the need for scaling both negative and positive poles of each dimension (e.g., annoying and pleasant; vibrant and monotonous, etc.). Where individual participant behavior might rely on several demographic, social, psychological, and affective variables and not be an easy problem to solve, systematic common behaviors across the population are easier to detect by analyzing general trends over the scores.

### 2.3. A Note on Terminology

Throughout this paper, the following terms are used in order to describe the survey data as collected and after the scaling method is applied:

Likert scale: The assessment scale relative to one perceptual attribute submitted to participants, comprising ordered categories that range from “strongly disagree” on one pole to “strongly agree” on the other.
Likert categories: The labels applied to the ordered categories (i.e., “strongly disagree,” “disagree,” “neither agree, nor disagree,” “agree,” “strongly agree”).
Likert scale metric: The geometrical function dictating the distances between the Likert points on one scale, ranging equal intervals between its points.
Likert value(s): The numerical value applied to each category (1–5, when considered as equidistant).
Rescaled metric: The new geometrical function dictating the distances between the Likert points of one scale, built to range perceptually equidistant intervals along different directions on the circumplex space.
Corrected value(s): The newly derived numerical values, based on the rescaled metric, to be applied to each Likert scale category.
ISO Pleasantness/Eventfulness or coordinates: The coordinate pair of values (x: Pleasant, y: Eventful) to place the response value on the bidimensional circumplex model.
Corrected ISO Pleasantness/Eventfulness or coordinates: The coordinate pair of response values on the circumplex model (x: Corrected Pleasant, y: Corrected Eventful) calculated through the rescaled metrics.

It has been noted that the term “metric” is inconsistently used and understood across fields and studies, where it may be interpreted as a statistic or index, as a synonym of “scale,” or to distinguish between ordinal (non-metric) and interval (metric) data (Adroher et al., 2018). In this paper, its use is intended as its mathematical definition, as a distance function.

## 3. Methodology

### 3.1. Soundscape Data Collection Method

**Figure 2:**
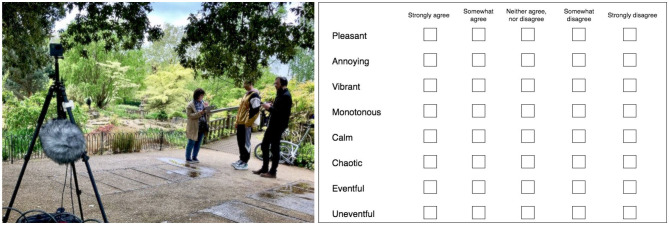
**(Left)** Data collection on site, public space user fills the questionnaire while extra visual and acoustics measurements are taken. **(Right)** Perceptual attributes scaling part of the questionnaire used during the data collection.

The data collection, currently used in some studies (Lionello et al., 2019; Aletta et al., 2020; Mitchell et al., 2020), followed the Soundscape Indices (SSID) Protocol (Mitchell et al., 2020), collecting *in situ* responses (soundscape assessment data) from users of public spaces at 11 different locations in London (UK) (Figure 2). For the soundscape-related questions, the SSID protocol is in turn based on Method A of the (ISO/TS, 2018). At each site, approximately 100 participants were asked to fill a questionnaire including the scaling of the eight perceptual attributes—i.e., pleasant, calm, uneventful, monotonous, annoying, chaotic, eventful, and vibrant—on a five-point Likert scale ranging from “strongly agree” (5) to “strongly disagree” (1) (see Figure 2). In order to reach the required amount of participants, surveys were collected during multiple sessions at the same location, trying to meet the same general context (e.g., time of the day, weather conditions, and social presence).

### 3.2. Participants

In addition to the soundscape-related questions, the SSID protocol also collected basic demographics about the participants. There is some evidence to suggest that personal characteristics such as age, gender, and educational level can influence a person's assessment of the soundscape (Yang and Kang, 2005; Xiao and Hilton, 2019) to a limited degree, however these factors have not been considered within this study. In total, the data collection included *N* = 984 respondents, comprising 52.9% female, 45.6% male, and 0.14% nonconforming or prefer-not-to-say, with a mean age of 34.7 years. Participants were required to be at least 18 years of age, but no maximum age limit was applied. The majority of the sample (57.5%) were full-time employed, with 3% unemployed, 7% retired, 32% student, and 6% other or rather-not-say. A plurality of respondents (36%) are university graduates, 1.9% have some high school, 15.7% are high school graduates, 12.9% have some college, 5% have some postgraduate work, and 23.2% have a postgraduate degree. According to data from Eurostat (Eurostat, 2020), the proportion of the Inner London working-age population who have completed university level or higher education was 66.8%, compared to 64.2% within this dataset, indicating a reasonable sampling of the local population. The self-identified ethnic composition was 70.3% white, 14.2% Asian/Asian British, 5.3% mixed/multiple ethnic groups, 2.7% Black/African/Caribbean/Black British, 1.9% Middle Eastern, 2% other ethnic group, and 2.6% rather not say, with 28.5% identifying as local, 27.8% tourist, 10.9% other, and 32.7% rather-not-say.

### 3.3. Data Collection Sites

Eleven urban public spaces were considered for data collection, with surveys occurring between March and October 2019 and including 30 total sessions. For each site (see Supplementary Figure 1), the initial goal was to collect 100 responses, meaning the data collection for a single site was often split over multiple sessions on successive days. A minimum number of 15 responses per session had been fixed to ensure consistency among the responses within a session. In most cases, due to incomplete questionnaires, restricted site access, and limited time, <100 responses per location were successfully selected for the final analysis. The total number of responses per site was as follows: Camden Town (CamdenTown: 94); Euston Tap (EustonTap: 98); Marchmont Community Garden (MarchmontGarden: 88); St Pancras Lock (PancrasLock: 90); Regent's Park Broadwalk (RegentsParkFields: 114); Regent's Park Japanese Garden (RegentsParkJapan: 90); Russell Square (RussellSq: 86); St Paul's Churchyard (StPaulsCross: 64); St Paul's Paternoster Row (StPaulsRow: 64); Tate Modern (TateModern: 100); Torrington Square (TorringtonSq: 96). More details about the sites can be found in Aletta et al. (2020).

The initial selection of investigated sites was driven by the need to include a reasonably varied sample of urban settings and contextual factors, including (but not limited to) urban morphology, architectural typology, dominant sound sources, amount of greenness, cultural/historical significance, and crowdedness. Due to the practicalities of performing large-scale *in situ* surveys (the most obvious of which is a minimum presence of members of the public to approach and invite for the survey), it was not possible to achieve a full spectrum of representative urban spaces types (e.g., surveying “semi-desert” public spaces is not possible if there are no people to approach). Consequently, the selected locations skew toward crowded urban squares, but do include a wide variety of greenness levels, visual openness, historical significance, and sound sources profiles. The resulting set of soundscape assessments therefore does not fully cover the soundscape circumplex space as defined by Axelsson et al. (2010), instead clustering toward the vibrant (i.e., positive pleasantness and positive eventfulness) quadrant. To some extent, this reflects an inherent challenge with conducting *in situ* data collection, as the accessible sites are limited by practical realities, a limitation which may only be possible to address in the future with further laboratory studies.

### 3.4. Data Processing

**Figure 3:**
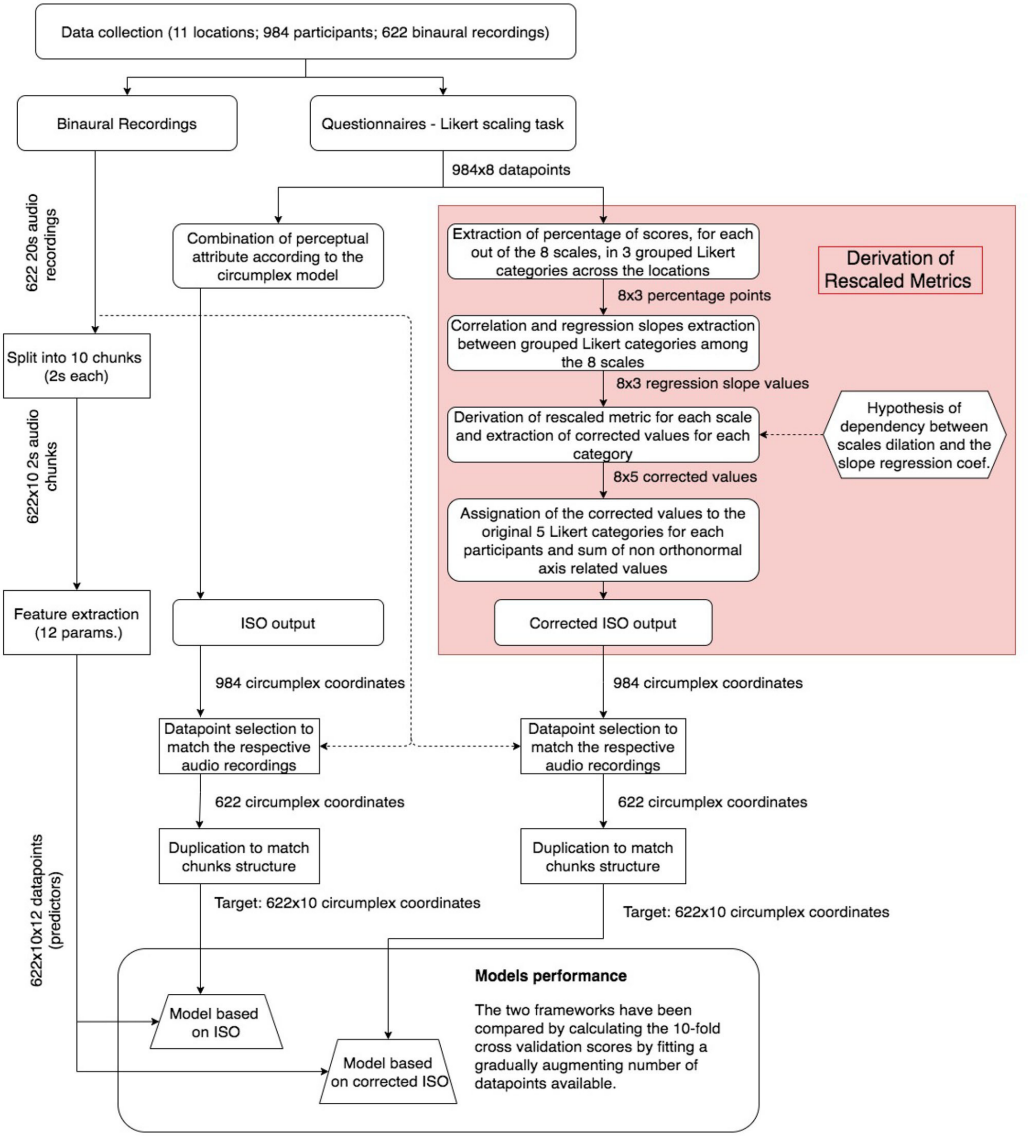
Flow chart of the methodology and data processing presented in this study.

An overall flowchart of how the data have been processed and used across the whole study is shown in Figure 3. Despite a dataset amounting to (*N* = 984) records, because of the lack of a homogeneous distribution across the five Likert categories and the relatively small number of total locations, weak correlations were initially found between single response categories. Thus, in order to investigate the interval properties of the Likert scale metrics, the scores of the Likert scales were collapsed into three grouped categories: “agreement” that included “strongly agree” and “somewhat agree” (1–2); “disagreement” that included “somewhat disagree” and “strongly disagree” (4–5); and “neutral” that corresponded to “neither agree nor disagree” (3) scores. This grouping choice was motivated by the need for leveling the distribution of the original categories, and for augmenting the precision of both correlation and slope regression analysis (which is introduced in section 3.4.2). This approach has also been adopted in previous studies on soundscape modeling (Giannakopoulos et al., 2019; Lionello et al., 2019). For each of the 11 locations, the percentage of scores (in terms of occurrences) falling in each group of these three new categories (agreement/neutral/disagreement) was calculated. Thus, 24 variables (3 categories * 8 perceptual attributes) for the 11 locations were considered.

#### 3.4.1. Slope Coefficients to Introduce Correction Factors

**Figure 4:**
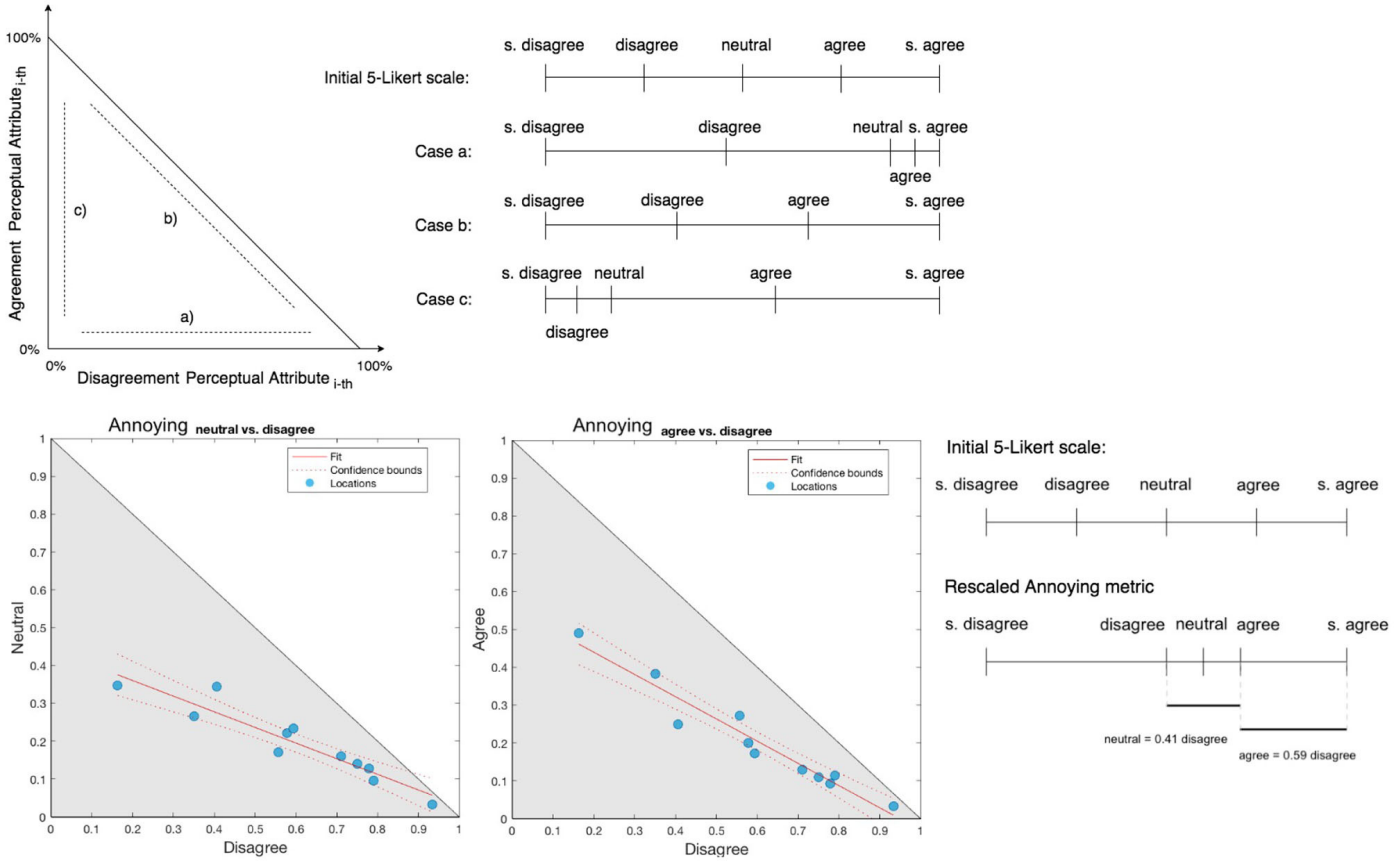
**(Top)** Boundary cases of regression slopes of percentage of agreement and disagreement grouped categories for a given perceptual attribute across multiple soundscapes. **(Bottom)** Instance of application of the rescaled on the annoying metric. The two regression slopes are then used to rescale coherently the metrics. The rescaled version of annoying metric, reported in this example, is different from the final version presented later in Figure 5 as, in this case, the metric is not enriched with information from the other scales.

In this section, the correlation of percentage of responses of grouped categories, found between the perceptual attributes across the 11 locations, were used to analyze a systematic behavior hidden in the way participants scaled their responses. The results of the analysis of these behaviors are then used in section 3.4.2 to calculate the new coefficients, which will be used in place of the original Likert scale values. By plotting the 11 soundscapes with respect to the ratio of scores falling between pairs of grouped categories, the soundscapes can range inside a triangular region bounded by y = -x+1 (in the boundary case of exact reciprocal proportions between the percentage of responses in the two grouped categories), y = 0, and x = 0 (in the boundary cases of no responses falling in one of the two categories examined, see Figure 4). Ideally, points would be expected to be randomly distributed within this region as the percentage of negative, positive, and neutral answers within each perceptual attribute are not expected to be correlated across the different soundscapes. Where these percentages are correlated, the points are not randomly distributed, and a regression slope coefficient can be derived from the pattern of points, as shown in Figure 4.

The procedure is based on the hypothesis that a dependency exists between the dilation or compression of the interval between two Likert categories and the regression slopes of percentage of answers falling in the respective grouped categories, which may either belong to the same scale or to two different scales. In this hypothesis, the interpretation of the dilation or compression of the Likert intervals is taken to be commonly shared across the participants, as it will be demonstrated in section 4.2.

To demonstrate this relationship, let us consider the following boundary cases in a scatter plot of soundscapes with respect to their percentages of agreements (dependent variable) and disagreements (independent variable) scores of an arbitrary perceptual attribute as seen in Figure 4. If the soundscapes lay onto a line with proportional coefficient equal to 0, the metric of the corresponding perceptual attribute would collapse across the disagreement poles ranging only between agreement and neutral values. In the case where the soundscapes lay onto a line with regression slope of −1, the percentage of agreement would be exactly reciprocal to the percentage of disagreement letting the number of the remaining neutral category scores be null. In this last case (case b in Figure 4), the neutral middle point score would be removed by making the whole scale range over 4 points instead of 5 points and by dilating the distances within both disagreement and agreement points.

By considering the previous examples, it is possible to advance the hypothesis that the angle β_*j*_ of the *j*th slope is linearly dependent on a dilation coefficient of the metric scale between the considered grouped Likert categories (see Figure 4). This allows us to introduce the following analysis of dilation across the intervals of the scale metrics.

#### 3.4.2. Metric Scale Dilation Between Perceptual Attributes

By examining the correlation between perceptual attributes, it is possible to obtain a description of the dilation and compression ratios between the metric scales belonging to the respective perceptual attributes. The slope regression between score categories from different perceptual attributes is used to set the proportions of the intervals across all the metric scales. However, one more assumption must be taken regarding the centering of the metrics. The current study can identify dilation and compression of the intervals, but this procedure still cannot properly identify an eventual shift between the scales.

Following the relationship between regression slopes and dilation factors introduced in section 4, the regression slopes were selected to link all the grouped categories across all the perceptual attributes by combining those coefficients associated with the largest correlation across all their possible combinations. Once the system of equations relating all the grouped categories to each other across all the perceptual attributes is obtained, it is then possible to calculate the proportion values between the grouped categories with respect to one of them, which is arbitrarily fixed. Once the three values for *a*_*i*,0_ (agree), *d*_*i*,0_ (disagree), and *n*_*i*,0_ (neutral) intervals for each perceptual attribute (*i* = 1, 2, …, 8) are obtained, the barycenter of each rescaled metric is set as $b_{i}=\frac{a_{i,0}+d_{i,0}}{2}$. Then in the new metric scale the “strongly agree” point is set to *aa*_*i*_ = *a*_*i*,0_ − *b*_*i*_ and the “strongly disagree” to *dd*_*i*_ = *d*_*i*,0_ − *b*_*i*_. The neutral is modeled as *n*_*i*_ = *z*_*i*_*b*_*i*_, where *z*_*i*_ ∈ {−1, 1} takes the sign according to the corresponding correlation between neutral and the other two grouped categories. The middle points “somewhat disagree” and “somewhat agree” are, respectively, found as *d*_*i*_ = *n*_*i*_ − *n*_*i*,0_/2 and *a*_*i*_ = *n*_*i*_ + *n*_*i*,0_/2. In three cases, namely vibrant, monotonous, and chaotic, no information was retrieved for *n*_*i*,0_ as the neutral score percentages did not score relevant correlation (either *r* < 0.7 or *p*> 0.05) with any other score category across the perceptual attributes. For these, an equal range interval was assumed between neutral point and each edge: $d_{i}=\frac{n_{i}+dd_{i}}{2}$ and $a_{i}=\frac{n_{i}+aa_{i}}{2}$. The Likert scale categories can now be assigned to the new values (*aa*_*i*_, *a*_*i*_, *n*_*i*_, *d*_*i*_, *dd*_*i*_|_*i* = 1, …, 8_) obtained for each category and each perceptual attribute. To calculate the new valence and arousal projection of one participant' assessments, all the new values assignation save for eventful and uneventful are summed together to calculate the valence, while annoying and pleasant are omitted and chaotic and calm changed of sign for the calculation of the arousal.

### 3.5. Application of the Rescaled Metrics

In order to test the usefulness of the correction factors, a classification model was built based on objective (psycho)acoustic metrics derived from 20-s binaural recording conducted while participants were responding to the survey. In order to compare the predictability of the two frameworks, the classification task was performed on both the ISO coordinates responses and with their corrected version. The models were designed to predict the individual assessments calculated as reported in section 3.4.2 and assigned to five categories (bins) defined by five equidistant intervals along the continuum of output values (number of samples falling in each bin in the corrected ISO coordinates pleasantness: [27, 144, 249, 134, 69], eventfulness: [33, 156, 304, 98, 32]). The same classification task was performed on the orthonormal projection by using the same predictors and samples (number of samples falling in each bin in the ISO coordinates pleasantness: [17, 107, 149, 240, 110]; eventfulness: [15, 139, 288, 145, 36]). The predictors, namely A-weighted sound level (LAeq), psychoacoustic loudness, sharpness, roughness, tonality, and speech interference level, were selected partially according to the results obtained across the soundscape modeling literature (Lionello et al., 2020) and calculated with the ArtemiS Suite software (v. 11.5, HEAD acoustics GmbH) (see Supplementary Material). The dataset used for this part partially overlaps what used to compute the correction values. Note that 622 binaural recordings taken during each filling of the questionnaires were cut to 20 s and split into 10 chunks 2 s long each. For each chunk, the mean and standard deviation of the previous listed acoustic parameters (see Supplementary Table 2) were calculated and used as input for the model. The models were fit for each of the four targets (ISO circumplex pleasantness and eventfulness and their corrected versions), multiple times with an increasing number of samples at each time to identify the convergence between training and validation data in the two systems of coordinates. Validation and training sets were composed of a total 622 × 10 datapoints by keeping all the chunks of one corresponding binaural recording on the same set. A 10-fold cross-validation algorithm performing Ridge Classification with Scikit-learn library for Python was performed on the progressively increasing number of samples passed to the model.

## 4. Results

### 4.1. Dependencies Within Paired Perceptual Attributes

Table 1 reports the correlations and regression slopes of the scores averaged for each location between only opposite perceptual attributes. Correlation between nonparallel perceptual attributes was not investigated as the correlation would follow the distribution of soundscapes across the circumplex model.

**Table 1:** Correlation and regression slopes between average scaling values of paired perceptual attributes across the 11 sites (e.g., annoying = −0.87 × pleasant + 5.51).

	**Correlation**	**Intercept**	**Slope**
Annoying vs. pleasant	− 0.99	5.51	− 0.87
Monotonous vs. vibrant	− 0.53	4.81	− 0.71
Chaotic vs. calm	− 0.99	5.28	− 0.81
Uneventful vs. eventful	− 0.79	4.67	− 0.65

*See Supplementary Figure 2 for the plotting*.

Annoying–pleasant and chaotic–calm pairs show similar results. In both cases, the range of mean values across the locations occupies a moderately large portion of the Likert scales (see Supplementary Figure 2 and Supplementary Table 1). In both cases, the linear dependency between the scores in the two pairs are characterized with large correlations (*r* = 0.99) and with slope coefficients with absolute values slightly lower than 1 (−0.87 for annoying–pleasant pair and −0.81 for chaotic–calm pair, where a −1 slope along with a +5 intercept value identifies a perfect overlap of two scales). The corresponding regression slopes show a larger agreement in the positive attribute (pleasant and calm) than disagreement for the negative attribute (annoying and chaotic). In the calm–chaotic pair, which scores are larger spread along the disagreement pole of calm and agreement pole of chaotic, it is also seen that a larger disagreement in calm corresponds to a smaller agreement in chaotic. The monotonous–vibrant pair shows a more random behavior (*r* = −0.53) with all their respective average scores falling in a small region close to neutral score (see also Supplementary Table 1) between neutral and somewhat agree for vibrant and between neutral and somewhat disagree for monotonous. Within the uneventful–eventful pair, despite a similar small range of values falling between neutral and somewhat agree for eventful (save for Marchmont Garden and Regents Park Fields locations, see Supplementary Table 1) and between neutral and somewhat disagree for uneventful, a moderate correlation (*r* = −0.79) indicates that participants are more likely to disagree with uneventful rather than agree with eventful.

### 4.2. Extraction of the Correction Values

For each location, the percentage of assessments falling in each of the three grouped categories (see section 3.4) was calculated in each observed location. Their correlation and p-values across all the locations were calculated between all the perceptual attributes and reported in Table 2. Regression slopes are shown in Table 3. In Table 2, it can be noticed that for each perceptual attribute the percentage of neutral scores are negatively correlated with agreement across positive perceptual attributes (pleasant, vibrant, calm, eventful), and negatively correlated with disagreement across negative perceptual attributes.

**Table 2:** Correlation table among percentage of agreement, disagreement, and neutral scaling for each perceptual attribute across all the soundscapes.

		**Pleasant**			**Annoying**			**Vibrant**			**Monotonous**			**Calm**			**Chaotic**			**Eventful**			**Uneventful**		
		**Disagree**	**Neutral**	**Agree**	**Disagree**	**Neutral**	**Agree**	**Disagree**	**Neutral**	**Agree**	**Disagree**	**Neutral**	**Agree**	**Disagree**	**Neutral**	**Agree**	**Disagree**	**Neutral**	**Agree**	**Disagree**	**Neutral**	**Agree**	**Disagree**	**Neutral**	**Agree**
Pleas.	Disagree	–	–	–	–	–	–	–	–	–	–	–	–	–	–	–	–	–	–	–	–	–	–	–	–
	Neutral	0.63^*^	–	–	–	–	–	–	–	–	–	–	–	–	–	–	–	–	–	–	–	–	–	–	–
	Agree	−0.96^***^	−0.82^**^	–	–	–	–	–	–	–	–	–	–	–	–	–	–	–	–	–	–	–	–	–	–
Ann.	Disagree	−0.92^***^	−0.85^**^	0.98^***^	–	–	–	–	–	–	–	–	–	–	–	–	–	–	–	–	–	–	–	–	–
	Neutral	0.77^**^	0.92^***^	−0.89^***^	−0.94^***^	–	–	–	–	–	–	–	–	–	–	–	–	–	–	–	–	–	–	–	–
	Agree	0.97^***^	0.73^*^	−0.97^***^	−0.97^***^	0.83^**^	–	–	–	–	–	–	–	–	–	–	–	–	–	–	–	–	–	–	–
Vib.	Disagree	0.64^*^	0.42	−0.62^*^	−0.67^*^	0.61^*^	0.66^*^	–	–	–	–	–	–	–	–	–	–	–	–	–	–	–	–	–	–
	Neutral	−0.10	−0.24	0.16	0.12	−0.27	0.00	−0.14	–	–	–	–	–	–	–	–	–	–	–	–	–	–	–	–	–
	Agree	−0.39	−0.11	0.33	0.39	−0.23	−0.48	−0.61^*^	−0.69^*^	–	–	–	–	–	–	–	–	–	–	–	–	–	–	–	–
Mon.	Disagree	−0.82^**^	−0.58	0.81^**^	0.82^**^	−0.76^**^	−0.8^**^	−0.55	0.00	0.40	–	–	–	–	–	–	–	–	–	–	–	–	–	–	–
	Neutral	0.08	0.03	−0.07	−0.12	0.10	0.12	0.13	0.54	−0.52	−0.52	–	–	–	–	–	–	–	–	–	–	–	–	–	–
	Agree	0.91^***^	0.66^*^	−0.91^***^	−0.89^***^	0.83^**^	0.86^***^	0.56	−0.31	−0.16	−0.86^***^	0.02	–	–	–	–	–	–	–	–	–	–	–	–	–
Calm	Disagree	0.88^***^	0.8^**^	−0.93^***^	−0.91^***^	0.87^***^	0.87^***^	0.43	−0.34	−0.03	−0.73^*^	−0.04	0.88^***^	–	–	–	–	–	–	–	–	–	–	–	–
	Neutral	0.10	0.73^*^	−0.33	−0.46	0.62^*^	0.30	0.24	0.03	−0.20	−0.13	0.03	0.13	0.31	–	–	–	–	–	–	–	–	–	–	–
	Agree	−0.84^**^	−0.87^***^	0.93^***^	0.93^***^	−0.92^***^	−0.87^***^	−0.44	0.32	0.06	0.71^*^	0.04	−0.85^**^	−0.99^***^	−0.44	–	–	–	–	–	–	–	–	–	–
Cha.	Disagree	−0.83^**^	−0.88^***^	0.92^***^	0.95^***^	−0.94^***^	−0.89^***^	−0.47	0.20	0.18	0.74^**^	−0.09	−0.82^**^	−0.95^***^	−0.54	0.98^***^	–	–	–	–	–	–	–	–	–
	Neutral	−0.18	0.24	0.05	−0.08	0.22	−0.02	−0.19	0.12	0.04	−0.07	0.27	−0.08	0.04	0.60^*^	−0.13	−0.27	–	–	–	–	–	–	–	–
	Agree	0.91^***^	0.84^**^	−0.97^***^	−0.96^***^	0.91^***^	0.93^***^	0.55	−0.25	−0.20	−0.75^**^	0.01	0.87^***^	0.98^***^	0.38	−0.98^***^	−0.96^***^	−0.02	–	–	–	–	–	–	–
Eve.	Disagree	−0.02	−0.03	0.03	−0.02	−0.07	0.08	0.33	0.67^*^	−0.78^**^	−0.15	0.56	−0.16	−0.36	0.16	0.32	0.19	0.06	−0.21	–	–	–	–	–	–
	Neutral	−0.67^*^	−0.70^*^	0.74^**^	0.70^*^	−0.64^*^	−0.70^*^	−0.54	0.19	0.24	0.43	−0.06	−0.47	−0.71^*^	−0.33	0.72^*^	0.70^*^	0.20	−0.79^**^	0.07	–	–	–	–	–
	Agree	0.41	0.44	−0.46	−0.41	0.43	0.36	0.07	−0.63^*^	0.45	−0.14	−0.39	0.4	0.7^*^	0.07	−0.68^*^	−0.56	−0.17	0.63^*^	−0.8^**^	−0.65^*^	–	–	–	–
Uneve.	Disagree	−0.07	0.08	0.02	0.06	0.05	−0.13	−0.32	−0.57	0.69^*^	0.09	−0.14	−0.02	0.30	−0.10	−0.27	−0.18	0.06	0.17	−0.81^**^	−0.30	0.80	–	–	–
	Neutral	−0.46	−0.61^*^	0.55	0.58	−0.70^*^	−0.45	−0.49	0.51	−0.06	0.49	−0.17	−0.47	−0.67^*^	−0.35	0.68^*^	0.66^*^	−0.07	−0.67^*^	0.34	0.70^*^	−0.68*	−0.57	–	–
	Agree	0.42	0.34	−0.43	−0.50	0.45	0.49	0.74^**^	0.33	−0.8^**^	−0.46	0.29	0.37	0.11	0.37	−0.16	−0.26	−0.03	0.27	0.74^**^	−0.15	−0.48	−0.8^**^	−0.03	–

* *p < 0.05*,

** *p < 0.01*,

*** *p < 0.001*.

**Table 3:** Regression slopes for correlation coefficients *r* > 0.7 and *p*-values *p* < 0.05.

		**Pleasant**			**Annoying**			**Vibrant**			**Monotonous**			**Calm**			**Chaotic**			**Eventful**			**Uneventful**		
		**Disagree**	**Neutral**	**Agree**	**Disagree**	**Neutral**	**Agree**	**Disagree**	**Neutral**	**Agree**	**Disagree**	**Neutral**	**Agree**	**Disagree**	**Neutral**	**Agree**	**Disagree**	**Neutral**	**Agree**	**Disagree**	**Neutral**	**Agree**	**Disagree**	**Neutral**	**Agree**
Pleas.	Disagree	–	–	–	–	–	–	–	–	–	–	–	–	–	–	–	–	–	–	–	–	–	–	–	–
	Neutral	–	–	–	–	–	–	–	–	–	–	–	–	–	–	–	–	–	–	–	–	–	–	–	–
	Agree	−0.74	−0.36	–	–	–	–	–	–	–	–	–	–	–	–	–	–	–	–	–	–	–	–	–	–
Ann.	Disagree	−0.81	−0.40	1.10	–	–	–	–	–	–	–	–	–	–	–	–	–	–	–	–	–	–	–	–	–
	Neutral	1.90	0.90	−2.5	−2.29	–	–	–	–	–	–	–	–	–	–	–	–	–	–	–	–	–	–	–	–
	Agree	1.33	0.68	−1.81	−1.65	0.74	–	–	–	–	–	–	–	–	–	–	–	–	–	–	–	–	–	–	–
Vib.	Disagree	–	–	–	–	–	–	–	–	–	–	–	–	–	–	–	–	–	–	–	–	–	–	–	–
	Neutral	–	–	–	–	–	–	–	–	–	–	–	–	–	–	–	–	–	–	–	–	–	–	–	–
	Agree	–	–	–	–	–	–	–	–	–	–	–	–	–	–	–	–	–	–	–	–	–	–	–	–
Mon.	Disagree	−1.72	–	2.32	2.14	−0.95	−1.30	–	–	–	–	–	–	–	–	–	–	–	–	–	–	–	–	–	–
	Neutral	–	–	–	–	–	–	–	–	–	–	–	–	–	–	–	–	–	–	–	–	–	–	–	–
	Agree	1.99	–	−2.69	–2.47	1.09	1.50	–	–	–	−1.18	–	–	–	–	–	–	–	–	–	–	–	–	–	–
Calm	Disagree	0.74	0.36	−1.0	−0.92	0.4	0.55	–	–	–	−0.46	–	0.38	–	–	–	–	–	–	–	–	–	–	–	–
	Neutral	-	2.32	–	–	–	–	–	–	–	–	–	–	–	–	–	–	–	–	–	–	–	–	–	–
	Agree	−0.70	−0.34	0.94	0.86	−0.37	−0.52	–	–	–	0.44	–	−0.36	−0.94	–	–	–	–	–	–	–	–	–	–	–
Cha.	Disagree	−0.88	−0.42	1.17	1.07	−0.47	−0.65	–	–	–	0.53	–	−0.45	−1.18	–	1.24	–	–	–	–	–	–	–	–	–
	Neutral	–	–	–	–	–	–	–	–	–	–	–	–	–	–	–	–	–	–	–	–	–	–	–	–
	Agree	0.89	0.44	−1.21	−1.10	0.49	0.67	–	–	–	−0.55	–	0.46	1.22	–	−1.29	−1.04	–	–	–	–	–	–	–	–
Eve.	Disagree	−	–	–	–	–	–	–	–	−1.11	–	–	–	–	–	–	–	–	–	–	–	–	–	–	–
	Neutral	–	−1.39	3.86	3.56	–	−2.14	–	–	–	–	–	–	−3.95	–	4.05	–	–	−3.11	–	–	–	–	–	–
	Agree	–	–	–	–	–	–	–	–	–	–	–	–	2.34	–	–	–	–	–	−0.78	–	–	–	–	–
Uneve.	Disagree	–	–	–	–	–	–	–	–	–	–	–	–	–	–	–	–	–	–	−1.07	–	1.41	–	–	–
	Neutral	–	–	–	–	−2.13	–	–	–	–	–	–	–	–	–	–	–	–	–	–	–	–	–	–	–
	Agree	–	–	–	–	–	–	1.05	–	−1.41	–	–	–	–	–	–	–	–	–	1.33	–	–	−1.25	–	–

*Independent variables are in the rows, and dependent variables are in the columns (e.g., pleasant_disagree_ = - 0.74 × pleasant_agree_+const.)*.

**Figure 5:**
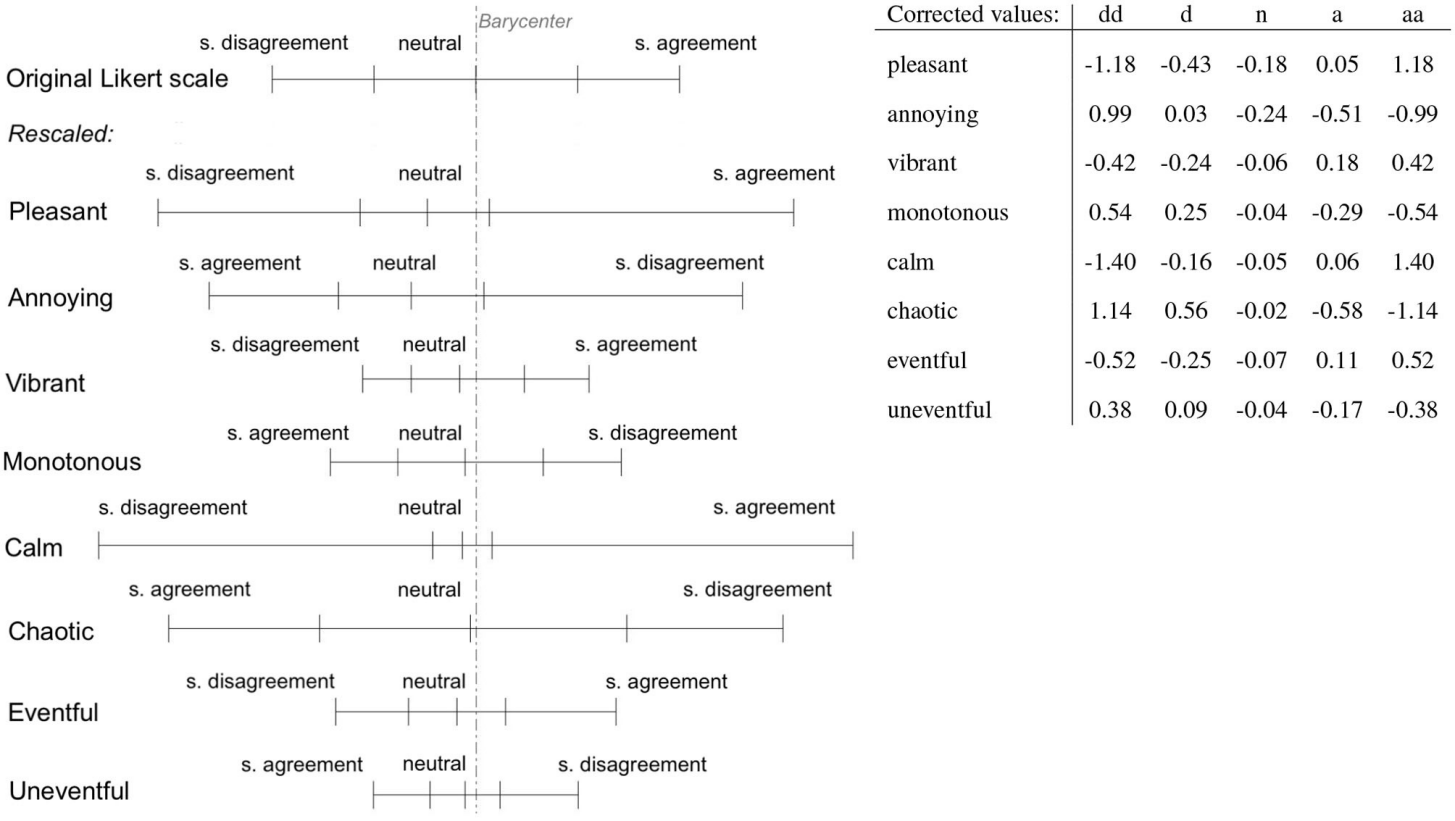
**(Left)** Graphical representation of the dilation and compression across the dimensions. **(Right)** Corrected values of the Likert scales. dd, strongly disagree; d, somewhat disagree; n, neither agree nor disagree; a, somewhat agree; aa, strongly agree.

The correction factors method introduced in section 3.4.2 has been applied to extract these values from Figure 3 and these are reported in numerical and visual format in Figure 5. The values, reported in the same figure, are not normalized as the method introduced provides a relative proportional information between the scales. The results obtained in the table are given by setting the pleasant disagree value *a*_0_ = −1, following the formulas introduced in section 3.4.2.

In-depth discussions of these results for each perceptual attribute have been included in Supplementary Material (section S.1).

### 4.3. Application on Soundscape Modeling

**Figure 6:**
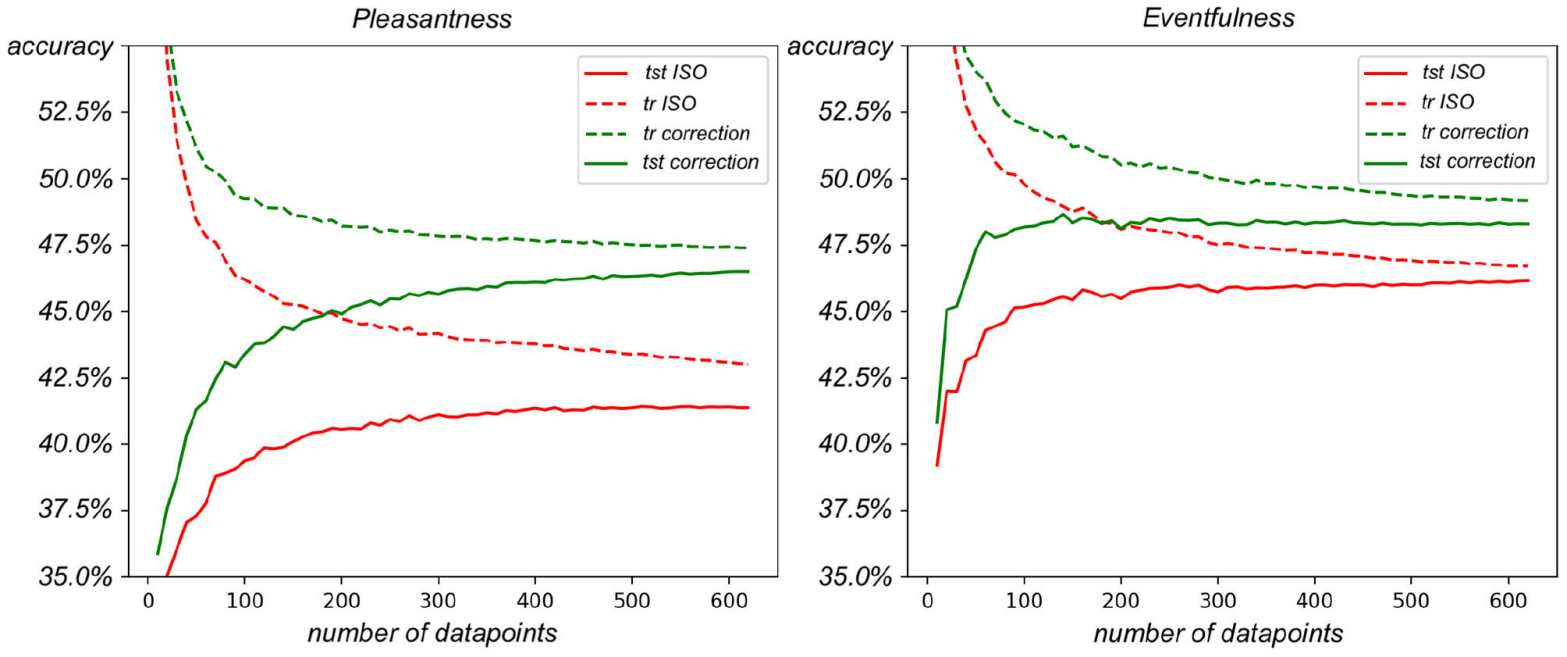
Comparison of Ridge Classifiers fit on the corrected and original version of the ISO circumplex coordinates while increasing the number of datapoints. **(Left)** ISO and corrected ISO circumplex pleasantness. **(Right)** ISO and corrected ISO circumplex eventfulness.

The results of the Ridge Classifier prediction models for predicting both the ISO targets and the rescaled metric targets are shown in Figure 6. For both the circumplex coordinates, the results show a higher accuracy on training and test sets for the model predicting the categories of re-scaled items compared to the one predicting the categories computed from the ISO-(accuracy of pleasantness on the rightmost point of the curve: ISO 41.4%, rescaled 46.5%; accuracy of eventfulness on the rightmost point of the curve: ISO 46.2%, rescaled 48.3%). Nonetheless, it can be observed from the graphs a closer convergence of the learning curve for the model based on the rescaled metrics rather than the original ISO one. The training curves reported in Figure 6 provides an upper limit under which the validation performance can improve. By augmenting the number of samples fit in the model, the training tests decrease their accuracy as they rely on a larger variance across the samples. At the same time, it is more likely that the statistics of samples in the test sets match the ones in the training sets, therefore increasing the performance of the test set. However, the distances between the training and test curves in each of the four targets (ISO pleasantness, corrected pleasantness, ISO eventfulness, and corrected eventfulness) show that there is still a margin of improvement for the current models, which can be achieved by augmenting the data samples. Nevertheless, the distances between the training curves, in both graphs, show a systematically better performance, according to the model framework used in this study, of the corrected coordinates compared to the ISO ones.

## 5. Discussion

### 5.1. Interpretation of the Correlations Within Pairs of Perceptual Attributes

The high correlation coefficients found in Table 1 suggests a systematic unbalanced interpretation of the scales within pairs of perceptual attributes. By plotting the regression slopes found from Table 1, given in Supplementary Figure 2, the following conclusions can be made. A general trend across the soundscapes in our dataset shows that the average participant tends to assess a given soundscape as more pleasant than it is not annoying. This pattern continues to the other side of the pole, where a soundscape is rated as relatively less annoying than it is not pleasant, however this behavior is not symmetrical about neutral. The whole line demonstrating this behavior is shifted toward pleasant, such that a neutral pleasant rating (3) on average corresponds to a slightly lower than neutral (2.9) annoying rating. These trends are replicated similarly for the perceptual attribute pair calm–chaotic. Despite this slight unbalance between pleasant to annoying and calm to chaotic ratings, strong correlations (*r* = −0.99 for both pairs pleasant–annoying and calm–chaotic) are still present, as shown in Table 1.

A possible explanation for the unbalanced patterns observed in some pairs of attributes is that, when performing the scaling task, participants indeed do not recognize and/or interpret them as being paired, or else, semantically opposite as per the circumplex framework. While for some cases the pairing may be more obvious (e.g., eventful–uneventful), one cannot assume this is always the case (e.g., vibrant–monotonous). Even so, when the circumplex space is not presented visually as such, it is difficult to confirm whether participants are detecting paired items as they could be associating different meanings to the attributes. Without the visual representation, the framework relies on a common understanding of the specific terms used in order to achieve the dimensional relationships. Respondents are presented with eight apparently unrelated perceptual attributes to score, and this could lead to some inconsistencies while scoring corresponding attributes.

### 5.2. Correlation on Percentage of Agree, Disagree, and Neutral Scores

**Figure 7:**
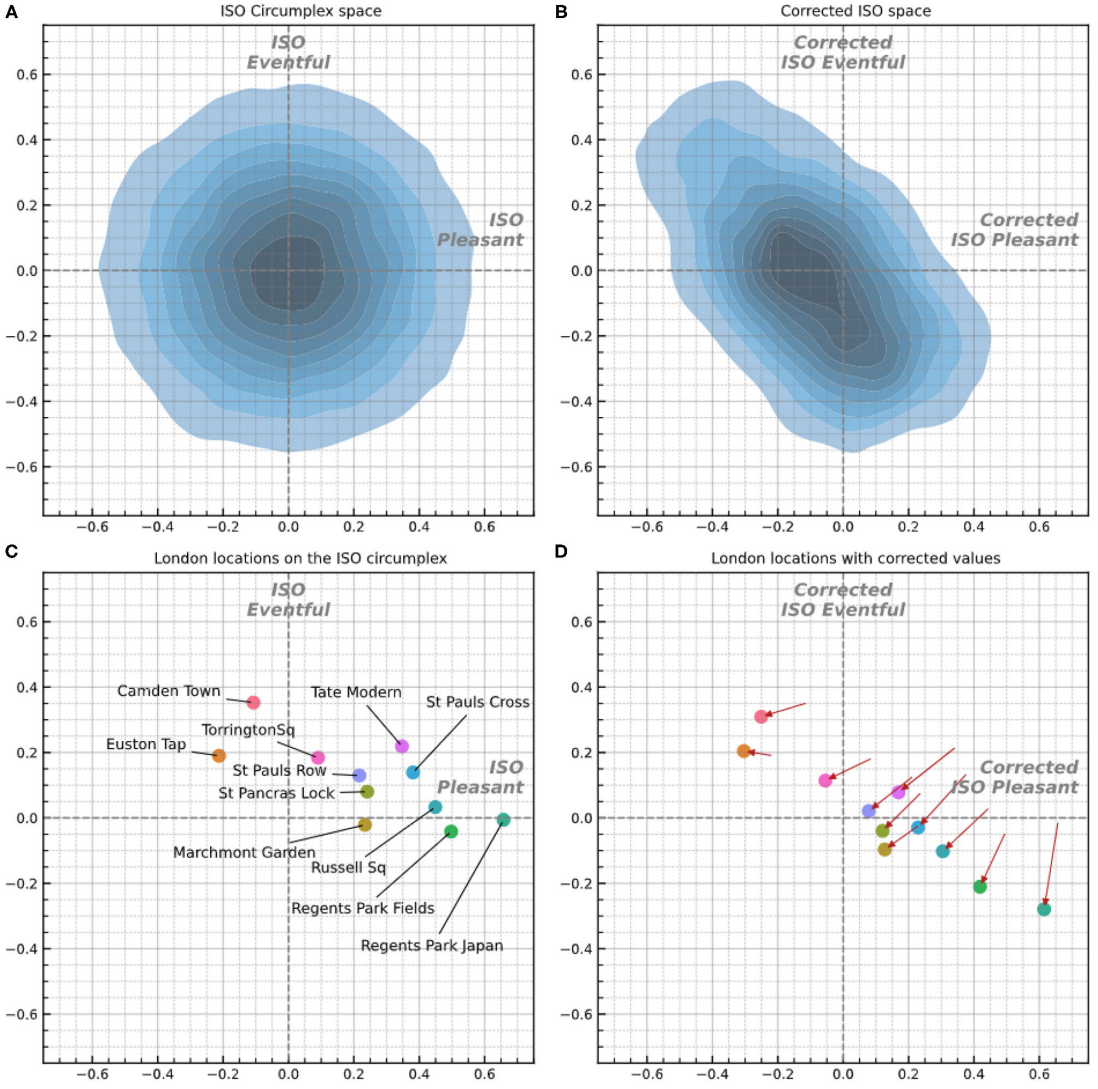
**(A)** Density plot of 30,000 randomly generated Likert responses projected on the circumplex model of soundscape according to the original metrics given by ISO ISO/TS (2019). **(B)** The same randomly generated responses corrected according to the values derived in section 3.4.2 and projected onto the corrected circumplex model. **(C)** London soundscape locations projected onto the ISO circumplex. **(D)** London soundscape locations after correction—arrows indicate the travel from their initial ISO coordinates to the new corrected coordinates. The x- and y-axes in all diagrams are normalized to [−1, 1].

It must be noticed from Figure 7 that the soundscapes are sampled from a narrow region parallel and transposed above the calm–chaotic bisector. A point which needs to be stressed is an eventual dependency between the distribution of the soundscapes onto the circumplex model and the results from Table 2. However, the low *p*-values in Table 2 suggests the hypothesis that the slopes could enclose some universal properties of the soundscapes and are not dependent on specific locations.

The random behavior expected between percentage of assessments falling in each categories across the locations (see section 3.4.1) is shown to be not assessed in Table 2. The neutral answers show, especially across pleasant and annoying in Table 3, high correlations with the other two groups of Likert categories. Other strong correlations can be seen in multiple slopes in Table 2. This unexpected results show that there is some systematic behavior in the percentage classes and so a systematic biased interpretation of the Likert scaling.

### 5.3. Projection Onto the Corrected Circumplex Space

To demonstrate and visualize the scaling effects across the soundscape circumplex space, a density plot with randomly generated data is shown in Figures 7A,B. Note that 30,000 responses were simulated for each of the eight perceptual attributes, with a uniform distribution of integers from 1 to 5, representing raw Likert scale responses that uniformly cover their respective axes. These were then projected according to the recommendations of ISO/TS 12913-3:2019 (as shown in Figure 1), resulting in a normal distribution of responses in both the ISO Pleasantness and Eventfulness axes, and the distribution density is plotted on the bidimensional axis (Figure 7A). This dataset is then scaled according to the correction values shown in Figure 5 and projected and normalized as described above. The resulting density distribution of the corrected circumplex space is plotted in Figure 7B. The change in the shape of the distribution density shown when moving from Figures 7A,B demonstrates the scaling of the original ISO space performed by the derived correction values.

Two main changes can be observed in the corrected density distribution: (1) an overall shift of the modal center of the space along the negative horizontal axis, and (2) a stretching along the chaotic–calm axis. In particular, this results in a compression of the vibrant-monotonous dimension—in practical terms, when this scaling is applied, soundscapes which may have fallen within the vibrant quadrant according to the standard method are likely to be resented as shifted toward the calm or chaotic quadrants. The compression found in Figure 7B shows a more likely representation of how the circumplex model is interpreted and experienced during the scaling task.

Moreover, this representation could be helpful in understanding distances between soundscapes and the actual impact in variation of coordinates when manipulating some elements in an existing or simulated soundscape, when visiting the same location under different contextual conditions, or when sampling assessments associated to participants with different perceptual sensitivity. Nonetheless, this change affecting the vibrant region of the model may also reflect a misunderstanding or disagreement about the meaning of vibrancy (as a perceptual construct) among respondents. This argument is partially supported by the results shown in Tables 1, 2, where significant correlations with the vibrancy attribute are limited to eventful and uneventful. This would be consistent with a previous study where “vibrant” was found to be correlated with “eventful” but not with “pleasant” (Aletta and Kang, 2018), which is generally in contrast with the theory underpinning the circumplex model of affect. Previous literature shows that vibrant soundscapes are associated with simultaneous social presence (e.g., human sounds of chatter or laughter) and presence of musical sounds. Such features were not necessarily typical at the 11 sampled locations, so this could have resulted in more scattered responses around the vibrancy construct, inflating their representation in the un-corrected ISO model.

This analysis of uniformly simulated response data also reveals some fundamental concerns with the ISO circumplex framework, outside of the metric interpretation addressed by the correction values. The fact that random data, which uniformly cover the initial perceptual attribute space, are then transformed to a normal distribution in the projected ISO circumplex space indicates that, contrary to the common interpretation, the circumplex space bounded by [−1, 1] is not uniformly available to be populated by soundscapes. This is a more fundamental question within the circumplex projection framework, which is independent of the Likert metric scaling caused by respondents' interpretations of the Likert scale, which is otherwise the focus of this study.

Taking the density distribution shown in Figure 7A as a probability density of where soundscapes can fall in the circumplex, it can be seen that soundscapes are much more likely to be placed toward the center of the circumplex. In this view, the effective limit for a soundscape composed of multiple responses (e.g., taken across a location) is in reality around [−0.6, 0.6], not [−1, 1]. Within the randomly generated data, <0.46% of pleasant values fell above 0.6. As such, extreme values on each of the perceptual dimensions are less likely to occur than are coordinate values, which place the soundscape in the neutral areas of the circumplex space. This means an extremely calm (or chaotic, or vibrant, etc.) coordinate is significantly less likely to occur than a neutral coordinate. The field of soundscape studies should therefore adjust our conception of the ISO circumplex space from ideally being equally populated by soundscapes across the full [−1, 1] and reframe our scaling of the value of the ideal “most pleasant” soundscape from [1, 0] to [0.6, 0]. Alternatively, a separate method of projecting and representing the pleasantness vs. eventfulness values, which does conform with the common understanding in the field, could be developed.

### 5.4. Correction of London Soundscape Coordinates

Applying the correction metrics to the actual London soundscapes data demonstrates how this compression and correction of the circumplex space affects the coordinates of real locations. Figure 7C shows the London soundscape locations projected into the ISO circumplex space, and in Figure 7D, the locations' corrected coordinates are plotted. The coordinates of each soundscape in the new circumplex model are determined by replacing the original scores of the assessments given by the participants—ranging from 1 “strongly disagree” to 5 “strongly agree”—with the new scores reported in Figure 5. The coordinates are then normalized ranging from [−1, 1] by dividing them by the sum of the positive scores reachable in both the corrected pleasant and eventful dimensions.

The comparison of the new projection with the one done through the original metric values becomes a complex task as the new dimensions lose some information such as the slopes of the diagonal sub-dimensions and the neutral assessment regions. The general movement of the soundscape coordinates (as indicated by the arrows in Figure 7D) reflect the transformation of the circumplex indicated in Figure 7B. “Regents Park Japan” appears to be the calmest and one of the most pleasant soundscapes (whose value does not seem to be much affected by the new metrics) and least eventful soundscape in the new metric. “Camden Town” maintains the same high value of pleasantness, and “Euston Tap” remains the soundscape with the lowest pleasantness score. “Russell Sq,” “St Pancras Lock,” and “St Pauls Cross” are shifted from the vibrant to the calm quadrant and “Torrington Sq” is moved from the vibrant to chaotic quarter. The eventfulness distance between “Torrington Sq” and “Euston Tap” is significantly increased as well as the distance between “Tate Modern” and “Euston Tap.” An overall trend appears to compress the distribution in a narrow region along the calm–chaotic bisector. Finally, it is possible to notice a compression along the pleasant dimension over those soundscapes falling in the positive pleasant side of the new projection plane, while the negative pleasant side of the plot preserves a similar spread as the original model.

### 5.5. Comparison of Linear Predictability Between ISO and Corrected ISO Targets

The better performance of Ridge Classifier introduced in section 4.3 in predicting the new metric compared to the ISO targets suggests a better linear mapping between acoustic information and the newly retrieved metrics compared to the raw orthonormal projection described in the standard. This performance improvement of the linear modeling task supports the idea that the corrected values create a better linear representation of the Likert scale, increasing the validity of applying mathematical operations that assume equidistant Likert categories. Specifically within soundscape studies, the improvement of the modeling results indicate that these correction values should be applied for the construction of future predictive soundscape models, which make use of the ISO circumplex framework. It should be noted that in this example the results are limited to a linear modeling case; it is unclear at this stage whether an eventual model that can incorporate nonlinear mapping would demonstrate the same improvement in performance using the rescaled metric values.

### 5.6. Limits of the Current Framework

As introduced earlier in section 1, the output of the correction scale model is bound by some constraints inherited by the design of the data collection. Here, follows a discussion upon these bonds trying to answer what it is expected to happen when some of these conditions change. It is first assumed that the output of the model is not affected by the particular distribution of the locations across the perceptual attributes space. This assumption is first needed under the consideration that in spite of the relatively narrow distribution of the locations across their perceptual attributes, as shown by the projection onto the circumplex model, the amount of locations in relation to the number of participants in each site makes the current dataset one of the largest projects in soundscape data collection that uses *in situ* surveys. The missing regions on the circumplex model, not covered in the current study, represent situations where the collection of the data represents a challenging task because of the reduced number of potential participants either because of low density of persons in the areas or because of less likely attitude to participating to the study. For the locations corresponding to these cases, the data collection is arranged to be performed through laboratory experiments. However, the biases introduced by the environmental validity would not fit the requirements of the current study. In those studies where extra laboratory experiments are required to fill missing regions across the score distributions of the scales, these are expected to be analyzed in comparison to the ground truth output of the model derived directly from the target collection procedure.

Our results and previous literature indicate that there is uncertainty around the concepts of vibrancy and monotonous within the ISO soundscape standard. This method has attempted to address some of this uncertainty internally, however it may also be possible to partially address this at the data collection stage by adjusting the semantic attributes used for these dimensions. This would then likely reduce the amount of internal correction needed. It is worthwhile to remark that in the original Swedish Soundscape Quality Protocol (SSQP) developed by Axelsson et al. (2010), the attribute used was “exciting,” which was their translation from the Swedish version of the questionnaire. Starting with Cain et al. (2013), this was replaced with “vibrant,” which has made its way into the ISO standard version. Future work in the space should investigate the differences between these and other versions of the attributes on the vibrant/monotonous dimension, as well as the usefulness of presenting multiple descriptions of the attributes to respondents.

## 6. Conclusions

When performing mathematical operations using Likert scaled survey data—whether that be calculating the mean of the scale values or performing a multidimensional projection—assumptions about the distance metric underlying the scale must be made. The typical assumption of equidistance between categories has been shown to not hold when examining multidimensional, paired Likert scales. By examining the correlations between response rates of the grouped Likert categories, and extracting commonly shared interpretations of the metric scaling, corrected Likert values are calculated. These corrected values account for the lack of correspondence between the equidistant Likert metrics and the participants' actual interpretation of the scaling task, thereby allowing mathematical operations to be valid when applied to the data. The implications of this scaling have been demonstrated through a Linear Ridge Classifier task, which shows significant improvement when applied to the corrected data.

This study was conceived and developed in the context of soundscape standardization processes about data collection methods and data analysis. The identity map that should match the interpretation of the scaling task for public space users with the formal model was questioned. Participants in the study used the scales differently from what would be expected based on the soundscape assessment theoretical framework. To address this, a correction factor matrix has been introduced for adjusting the Likert scale metrics and extracting corrected values applied to the categories for each Likert scale.

The findings indicate that (1) in soundscape studies, intervals are not necessarily interpreted to range equidistant spaces between Likert scale categories; (2) there is a matching between neutral and disagreement assessment for positive soundscape attributes and a correlation between agreement and neutral assessments across negative soundscape attributes; (3) intervals centered on neutral assessments are generally interpreted to be smaller than intervals placed on the extreme of the scales; and (4) the new metric is better described by (psycho)acoustic features compared to the original Likert scale metric, when used as indicators to predict how people experience urban soundscapes.

Moreover, from the results and comparison of the two projected spaces, the ISO and the corrected one, the following points have been found. The ISO circumplex model framework implies that a perceptual shift in the bidimensional space is direction independent. In other words, when the soundscape of a location changes due to dynamics of its contextual, physical, or other variables, the magnitude of perceptual differences should be equal regardless of the direction of shift or initial position in the bidimensional space. However, our data show that this is not the case in the original ISO space. The lack of this position- and direction-independence property in the perception of the ISO circumplex model along with the lack of overlapping match between Likert categories belonging to different perceptual attributes makes the circumplex projection, as described in the current ISO/TS 12913-3:2019, less effective in describing soundscapes by means of pleasantness and eventfulness coordinates. Particularly, the ISO space is found to be effected by a dilation along the vibrant–monotonous dimension, in terms of participants' scaling behaviors along that direction and in comparison to the same spatial shift in other directions. This exaggerated stretch is due to the artifacts of the misleading assumption of equally ranged Likert intervals, which is then passed to the ISO projections. Therefore, the vibrant dimension is overestimated in its length, compared to the other directions, due to the artifacts inherited from the unbalanced Likert scales belonging to different perceptual attributes.

It has also been shown that uniformly sampled Likert values, unaffected by the metric interpretation otherwise discussed here, are projected into the raw ISO space as a normal distribution, as opposed to a uniform distribution. This fact implies that soundscapes cannot be fairly distributed across the whole of the available range. This means that the original ISO mapping of the perceptual attributes into the circumplex model is neither a good representation of participants' interpretation of the projected space, nor a meaningfully spread representation of different soundscapes.

These findings suggest that the current ISO standard suffers from some inaccuracies of the standard metric as it is inherited from the raw Likert categories. By implementing the procedure described in this study, soundscape studies would benefit from a better representation in terms of how listeners experience soundscapes. In the proposed corrected circumplex projection, the space metric is intended to provide a perceptually equally spread space, along all the perceptual attribute directions, based on the scaling patterns retrieved from the participants' responses.

## Data Availability Statement

The datasets presented in this article are not readily available because, data used for the study is part of a larger dataset currently under development. Requests to access the datasets should be directed to: kang@ucl.ac.uk.

## Ethics Statement

The studies involving human participants were reviewed and approved by Departmental approval by the Ethics Lead at the UCL Institute for Environmental Design and Engineering (BSEER Research Ethics—Low Risk Application). The patients/participants provided their written informed consent to participate in this study.

## Author Contributions

ML: conceptualization, methodology, validation, and investigation. ML, FA, and AM: formal analysis, visualization, resources, and writing. ML and AM: software and data curation. ML, FA, AM, and JK: discussion. FA and JK: supervision. JK: project administration and funding acquisition. All authors contributed to the article and approved the submitted version.

## Conflict of Interest

The authors declare that the research was conducted in the absence of any commercial or financial relationships that could be construed as a potential conflict of interest.

## References

[B1] Adroher N. D. Prodinger B. Fellinghauer C. S. Tennant A. (2018). All metrics are equal, but some metrics are more equal than others: a systematic search and review on the use of the term “metric.” PLoS ONE 13:e193861. 10.1371/journal.pone.0193861 29509813 PMC5839589

[B2] Aletta F. Brambilla G. Maffei L. Masullo M. (2017). Urban soundscapes: characterization of a pedestrian tourist route in Sorrento (Italy). Urban Sci. 1:4 10.3390/urbansci1010004

[B3] Aletta F. Guattari C. Evangelisti L. Asdrubali F. Oberman T. Kang J. (2019). Exploring the compatibility of “method a” and “method b” data collection protocols reported in the iso/ts 12913-2:2018 for urban soundscape via a soundwalk. Appl. Acoust. 155, 190–203. 10.1016/j.apacoust.2019.05.024

[B4] Aletta F. Kang J. (2018). enTowards an urban vibrancy model: a soundscape approach. Int. J. Environ. Res. Publ. Health 15:1712. 10.3390/ijerph15081712 30103394 PMC6122032

[B5] Aletta F. Oberman T. Mitchell A. Tong H. Kang J. (2020). Assessing the changing urban sound environment during the COVID-19 lockdown period using short-term acoustic measurements. Noise Mapp. 7, 123–134. 10.1515/noise-2020-0011

[B6] Axelsson Ö. Nilsson M. Berglund B. (2010). A principal components model of soundscape perception. J. Acoust. Soc. Am. 128, 2836–2846. 10.1121/1.3493436 21110579

[B7] Berglund B. Nilsson M. Axelsson Ö. (2007). “Soundscape psychophysics in place,” in Proceeding of InterNoise, Vol. 6, 3704–3711.

[B8] Cain R. Jennings P. Poxon J. (2013). The development and application of the emotional dimensions of a soundscape. Appl. Acoust. 74, 232–239. 10.1016/j.apacoust.2011.11.006

[B9] Carifio J. Perla R. (2008). Resolving the 50-year debate around using and misusing likert scales. Med. Educ. 42, 1150–1152. 10.1111/j.1365-2923.2008.03172.x 19120943

[B10] [Dataset] Eurostat (2020). Tertiary Educational Attainment, Age Group 25-64 by Sex and Nuts 2 Regions. Available online at: https://ec.europa.eu/eurostat/tgm/table.do?tab=table (Retrieved September 02, 2020).

[B11] Fiebig A. Herweg A. (2017). “The measurement of soundscapes -a study of methods and their implications,” in Internoise (Hong Kong).

[B12] Giannakopoulos T. Orfanidi M. Perantonis S. (2019). “Athens urban soundscape (athus): a dataset for urban soundscape quality recognition,” in International Conference on Multimedia Modeling (Thessaloniki).

[B13] ISO 12913-1:2014 (2014). Acoustics “Soundscape” Part 1: Definition and Conceptual Framework. Geneva: Standard, International Organization for Standardization.

[B14] ISO/TS 12913-2:2018 (2018). Acoustics “Soundscape” Part 2: Data Collection and Reporting Requirements. Geneva: Standard, International Organization for Standardization.

[B15] ISO/TS 12913-3:2019 (2019). Acoustics “Soundscape” Part 3: Data Analysis. Geneva: Standard, International Organization for Standardization.

[B16] Jamieson S. (2005). Likert scales: How to (ab) use them. Med. Educ. 38, 1217–1218. 10.1111/j.1365-2929.2004.02012.x 15566531

[B17] Kang J. Aletta F. Margaritis E. Yang M. (2018). A model for implementing soundscape maps in smart cities. Noise Mapp. 5, 46–59. 10.1515/noise-2018-0004

[B18] Kogan P. Turra B. Arenas J. Zeballos F. Hinalaf M. Perez Villalobo J. (2016). “Application of the swedish soundscape-quality protocol in one european and three latin-american cities,” in The International Conference on Acoustics (Buenos Aires).

[B19] Lantz B. (2013). Equidistance of likert-type scales and validation of inferential methods using experiments and simulations. Electron. J. Bus. Res. Methods 11, 16–28.

[B20] Likert R. (1932). A technique for the measurement of attitudes. Arch. Psychol. 140, 1–55.

[B21] Lindburg P. Friberg A. (2016). Personality traits bias the perceived quality of sonic environments. Appl. Sci. 6:405 10.3390/app6120405

[B22] Lionello M. Aletta F. Kang J. (2019). “On the dimension and scaling analysis of soundscape assessment tools: a casestudy about the "method a" of iso/ts 12913-2:2018,” in The 23rd International Congress on Acoustics (Aachen). 10.18154/RWTH-CONV-239311

[B23] Lionello M. Aletta F. Kang J. (2020). A systematic review of prediction models for the experience of urban soundscapes. Appl. Acoust. 170:107479 10.1016/j.apacoust.2020.107479

[B24] Maffiolo V. Castellengo M. Dubois D. (1999). Is pleasantness for soundscapes dimensional or categorical? J. Acoust. Soc. Am. 105:943 10.1121/1.425718

[B25] Mitchell A. Oberman T. Aletta F. Erfanian M. Kachlicka M. Lionello M. (2020). The soundscape indices (SSID) protocol: a method for urban soundscape surveys-questionnaires with acoustical and contextual information. Appl. Sci. 10:2397 10.3390/app10072397

[B26] Pell G. (2005). Use and misuse of likert scales. Med. Educ. 39, 970–970. 10.1111/j.1365-2929.2005.02237.x 16150039

[B27] Posner J. Russell J. Peterson B. (2005). The circumplex model of affect: an integrative approach to affective neuroscience, cognitive development, and psychopathology. Dev. Psychopathol. 17, 715–734. 10.1017/S0954579405050340 16262989 PMC2367156

[B28] Rickards G. Magee C. Artino A. (2012). You can't fix by analysis what you've spoiled by design: developing survey instruments and collecting validity evidence. J. Grad. Med. Educ. 4, 407–410. 10.4300/JGME-D-12-00239.1 24294413 PMC3546565

[B29] Russell J. (1980). A circumplex model of affect. J. Pers. Soc. Psychol. 39, 1161–1178.26804575

[B30] Xiao J. Hilton A. (2019). An investigation of soundscape factors influencing perceptions of square dancing in urban streets: a case study in a county level city in China. Int. J. Environ. Res. Publ. Health 16:840. 10.3390/ijerph16050840 30866579 PMC6427759

[B31] Yang W. Kang J. (2005). Soundscape and sound preferences in urban squares: a case study in sheffield. J. Urban Design 10, 61–80. 10.1080/13574800500062395

