# Supplementary material for: Introducing a Method for Intervals Correction on Multiple Likert Scales: A Case Study on an Urban Soundscape Data Collection Instrument
Source: Front Psychol. 2021 Jan 22;11:602831. doi: 10.3389/fpsyg.2020.602831 (PMC7862575; doi:10.3389/fpsyg.2020.602831)
Supplement: Supplementary file 1 [file Data_Sheet_1.pdf]

Supplementary Material

1 ANALYSIS OF CORRELATION PATTERNS ALONG INDIVIDUAL SCALES

Here follows an analysis of correlation and regression slopes found across the individual scales from Tables 2 and 3. The outcome of this analysis has not been included, at the current stage, in the retrieval of the actual correction values, leaving this aspect for future possible improvement of the metric scales.

Pleasant

A strong negative correlation is observed between neutral and agreement across percentage of pleasant scores. Consequently, a strong positive correlation is observed between negative and neutral score percentage. These observations across all the soundscapes reveal a systematic, approximately, linear dependency between the percentages of participants who gave positive, or negative, and neutral scores. This suggests that the neutral score has been interpreted as shifted towards the negative side of the scale. By interpreting a low p-value in correspondence to correlation coefficient null to represent an equally ranged interval, the weak correlation between neutral and disagreement percentages scores, r = 0.63, suggests, by limiting the neutral interval to the disagreement side, that the intervals 'somewhat disagree'-'neutral' and the one between 'somewhat disagree'-'strongly disagree' show a less strong unbalance compared to the agreement side of the scale. The range of the intervals on the positive side of the scale is dictated by the regression slope between neutral (dependent variable) and agreement (independent variable) score percentage, m = -0.36, which indicates a larger interval interpreted by the participants across 'strongly agree'-'neutral' than across 'somewhat agree'-'somewhat disagree' along the pleasant scale. This is assumed by the observation that the boundary condition m = 0 would make the interval 'somewhat agree'-'somewhat disagree' collapse. The regression slope between agreement and disagreement, m = -0.74, suggests that the agreement interval is interpreted larger than the disagreement one. By comparing the previous two regression slopes it is also possible to deduct that the interval between disagreement edge and somewhat disagree is approximately twice the interval between somewhat disagree and somewhat agree. Thus, the pleasant metric, when considered alone and without using the information from the other scales, will show its neutral point falling on the disagreement side of the scale, a somewhat disagreement point placed about at two thirds between the disagreement edge and neutral point, and an agreement interval about a quarter larger than the disagreement interval.

Annoying

The annoying metric seems to be coherently symmetric to the pleasant metric shifting neutral score towards the agreement side with similar regression slopes (pleasant disagree against agree m = -0.74; annoying agree against disagree m = 1/(-1.65) = -0.61; pleasant neutral vs agree m = -0.36; annoying neutral vs disagree m = 1/(-2.29) = -0.44). A strong correlation appears between agreement and neutral, r = 0.83 (compare to pleasant disagreement and neutral r = 0.63). However, the regression coefficient is very close to 1 (m = 1.14) which still implies a very similar interval ranging interpretation over the agreement pole as it is for the disagreement pole in pleasant.

Vibrant, calm, chaotic, monotonous

Low correlation coefficients ($r \leq 0.7$) or absolute regressions slopes values close to 1, makes the interpretation of the intervals very uncertain by looking only at statistics within these single perceptual

values. Vibrant and calm show a negative correlation between neutral and agreement indicating that neutral scores tend towards the disagreement pole. Low correlation between disagreement and neutral in vibrant and between agreement and neutral in monotonous indicate equally ranged intervals across the disagreement side of the scale. In the calm and monotonous scales, slope coefficient values respectively of -0.94 and -1.18 between disagreement and agreement show a similar spread of intervals between the two sides of both scales.

Eventful

A moderate slope (m = -0.78) is found between disagree against agree suggesting, similarly to pleasant, a larger interval ranging across the agreement than across disagreement sides. As observed with monotonous, this fact follows the negative correlation between neutral and agree, indicating neutral tends towards the disagreement pole.

Uneventful

Similarly to what is observed with pleasant and annoying, the regression slope found in uneventful between agreement and disagreement is coherently reciprocal to the same pair in eventful.

2 CHARACTERISTICS OF THE LOCATIONS AND CORRELATION PLOTS

Location	Pleasant	Annoying	Vibrant	Monot.	Calm	Chaotic	Eventf.	Unevent.	ISO PL	ISO EVE
Camden Town	2.7 ± 1.3	3.0 ± 1.3	3.6 ± 1.2	2.7 ± 1.5	1.8 ± 1.1	3.7 ± 1.5	3.7 ± 1.2	2.2 ± 1.2	2.8 ± 0.6	3.7 ± 0.7
Euston Tap	2.5 ± 1.1	3.4 ± 0.9	3.1 ± 1.1	2.9 ± 1.4	2.0 ± 0.9	3.7 ± 1.0	3.2 ± 1.0	2.8 ± 1.3	2.6 ± 0.6	3.4 ± 0.6
Marchmont Garden	3.6 ± 1.5	2.4 ± 1.6	3.1 ± 1.0	2.4 ± 1.0	3.5 ± 1.4	2.7 ± 1.6	2.7 ± 1.2	2.8 ± 1.0	3.5 ± 0.9	3.0 ± 0.5
Pancras Lock	3.6 ± 1.1	2.3 ± 1.3	3.2 ± 1.1	2.3 ± 0.9	3.2 ± 1.5	2.7 ± 1.6	3.2 ± 1.1	2.7 ± 1.1	3.5 ± 0.8	3.2 ± 0.6
Regents Park Fields	4.3 ± 1.2	1.8 ± 1.3	3.4 ± 1.0	2.1 ± 1.1	4.0 ± 1.2	2.0 ± 1.4	2.9 ± 1.0	2.8 ± 1.1	4.0 ± 0.8	2.9 ± 0.5
Regents Park Japan	4.6 ± 0.7	1.4 ± 0.6	3.7 ± 1.1	2.0 ± 1.3	4.4 ± 0.8	1.6 ± 0.8	3.1 ± 1.2	2.4 ± 1.2	4.3 ± 0.6	3.0 ± 0.5
Russell Square	4.2 ± 0.6	1.8 ± 1.1	3.6 ± 0.8	2.2 ± 1.1	3.6 ± 1.0	2.3 ± 1.0	3.0 ± 0.9	2.7 ± 1.0	3.9 ± 0.6	3.1 ± 0.5
St. Pauls Cross	4.0 ± 0.7	2.0 ± 1.1	3.6 ± 0.8	2.2 ± 0.9	3.4 ± 1.5	2.5 ± 1.2	3.3 ± 1.2	2.4 ± 1.0	3.8 ± 0.6	3.3 ± 0.6
St. Pauls Row	3.7 ± 1.1	2.3 ± 1.3	3.5 ± 1.1	2.6 ± 1.3	3.0 ± 1.5	2.9 ± 1.4	3.2 ± 0.9	2.6 ± 1.1	3.4 ± 0.7	3.3 ± 0.6
Tate Modern	4.1 ± 0.6	2.1 ± 1.1	3.7 ± 0.9	2.0 ± 0.9	3.0 ± 1.6	2.7 ± 1.2	3.5 ± 0.8	2.3 ± 1.0	3.7 ± 0.6	3.4 ± 0.7
Torrington Square	3.3 ± 0.9	2.7 ± 1.2	3.5 ± 1.0	2.4 ± 1.3	2.6 ± 1.0	3.2 ± 1.1	3.2 ± 1.0	2.6 ± 1.2	3.2 ± 0.7	3.4 ± 0.7

Table S1. Mean ± standard deviation score for each perceptual attribute and ISO circumplex coordinates, normalized in this table to range between 1 to 5 similarly to what used in the Likert scales, across the locations (1 = strongly disagree, 5 = strongly agree).

Location	LAeq(dB)	N(sones)	R(asper)	SIL(dB)	S(acum)	T (tuHMS)
CamdenTown	68.1 ± 1.3	24.2 ± 2.2	0.047 ± 0.012	59.7 ± 1.3	2.11 ± 0.16	0.34 ± 0.21
EustonTap	67.3 ± 0.8	22.5 ± 1.3	0.039 ± 0.008	59.3 ± 0.8	2.11 ± 0.11	0.21 ± 0.14
MarchmontGarden	52.8 ± 1.2	8.5 ± 0.7	0.027 ± 0.006	44.6 ± 1.1	1.61 ± 0.14	0.13 ± 0.09
PancrasLock	57.8 ± 0.9	12.5 ± 0.7	0.030 ± 0.006	50.0 ± 0.8	1.92 ± 0.08	0.11 ± 0.08
RegentsParkFields	51.1 ± 1.4	7.5 ± 0.7	0.026 ± 0.007	42.5 ± 1.3	1.56 ± 0.15	0.13 ± 0.10
RegentsParkJapan	59.1 ± 0.7	15.2 ± 0.6	0.025 ± 0.005	52.0 ± 0.6	2.72 ± 0.10	0.09 ± 0.08
RussellSq	61.8 ± 0.8	17.2 ± 0.9	0.030 ± 0.006	54.1 ± 0.8	2.32 ± 0.09	0.16 ± 0.13
StPaulsCross	59.1 ± 1.2	12.6 ± 1.1	0.033 ± 0.008	50.6 ± 1.1	1.66 ± 0.13	0.14 ± 0.11
StPaulsRow	59.3 ± 1.3	12.6 ± 1.1	0.035 ± 0.008	50.3 ± 1.3	1.57 ± 0.14	0.18 ± 0.12
TateModern	59.6 ± 1.4	13.5 ± 1.2	0.033 ± 0.007	50.6 ± 1.4	1.59 ± 0.13	0.26 ± 0.17
TorringtonSq	59.7 ± 1.4	13.7 ± 1.4	0.034 ± 0.009	51.2 ± 1.3	1.81 ± 0.15	0.19 ± 0.13

Table S2. Overall acoustic statistics measured across the locations.

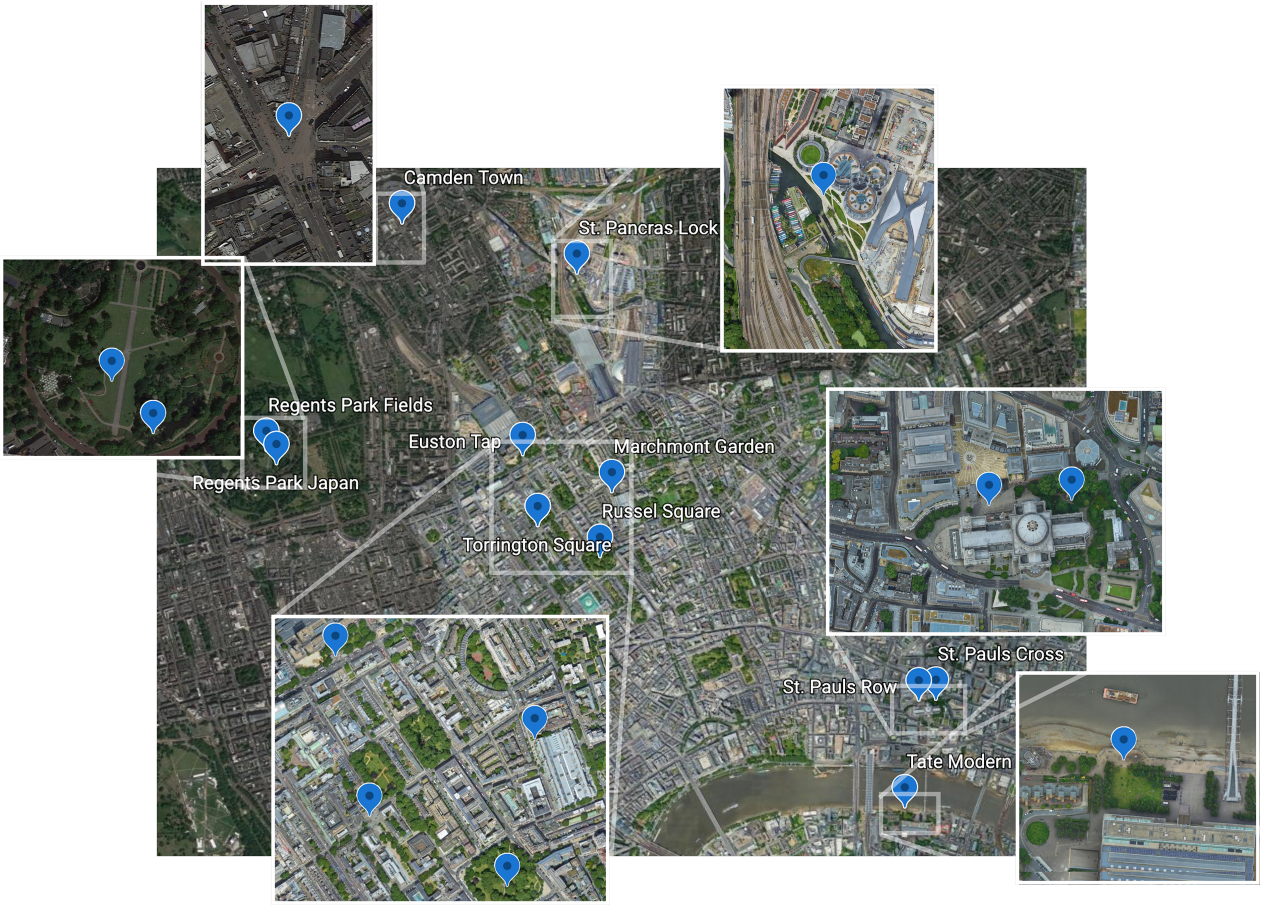

Figure S1. Maps of the 11 data collection sites across London, combination of labeled images taken from google earth

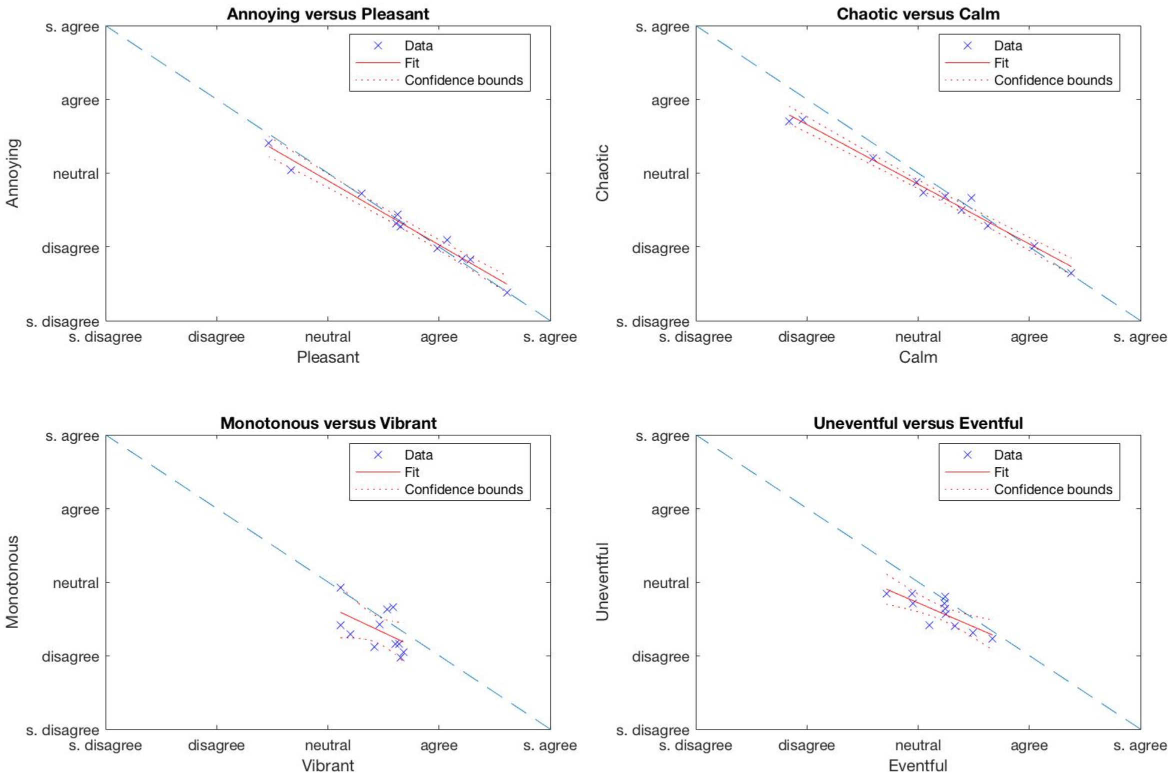

Figure S2. Plotting of the highly correlated pairs of attributes from Table 1. Left: Annoying against Pleasant average scores across the locations; Right: Chaotic against Calm average scores across the locations
